# Challenges of Estimating Accurate Prevalence of Arm Weakness Early After Stroke

**DOI:** 10.1177/15459683211028240

**Published:** 2021-07-28

**Authors:** Lisa A. Simpson, Kathryn S. Hayward, Moira McPeake, Thalia S. Field, Janice J. Eng

**Affiliations:** 1 Graduate Program in Rehabilitation Sciences, 8166 University of British Columbia, Vancouver, BC, Canada; 2 Departments of Physiotherapy, Florey Institute of Neuroscience and Mental Health, and NHMRC Centre of Research Excellence in Stroke Rehabilitation and Brain Recovery, University of Melbourne, Melbourne, VIC Australia; 3 Department of Physical Therapy, 574564 University of British Columbia, Vancouver, BC, Canada; 4 Neurosciences, 8167 Vancouver General Hospital, Vancouver, BC, Canada; 5 Division of Neurology, 8166 University of British Columbia, Vancouver, BC, Canada

**Keywords:** stroke, upper extremity, prevalence, rehabilitation

## Abstract

*Background*. Recent studies have reported lower statistics of upper limb (UL) weakness (48-57%) compared to widely cited values collected over 2 decades ago (70-80%). *Objective*. To explore potential factors contributing to the accuracy of prevalence values of UL weakness using a case study from a single regional centre. *Methods*. All patients admitted to the acute stroke unit with suspected diagnosis of stroke were screened from February 2016 to August 2017. Upper limb weakness was captured (a) prospectively using the Shoulder Abduction and Finger Extension (SAFE) score performed by unit physical therapists within 7 days post-stroke and (b) retrospectively via chart review using the National Institutes of Health Stroke Scale (NIHSS) arm score at admission and 24 hours post-admission. *Results*. A total of 656 patients were admitted with a first-ever stroke, and 621 (95%) individuals were administered the SAFE score. A total of 40% of individuals had UL weakness using the SAFE score (SAFE ≤8) at a mean time of 1.9 (SD 1.5) days post-stroke. In the same sample, 57% and 49% had UL weakness using the admission and 24-hour post-admission NIHSS arm score, respectively. *Conclusions*. The accuracy of population-level UL weakness prevalence values can be affected by weakness measure and score cut-off, time post-stroke weakness is captured, sample characteristics and use of single or multiple sites. Researchers using prevalence values for clinical trial planning should consider these attributes when using prevalence data for estimating recruitment rates and resource needs.

## Introduction

The most frequently cited statistic for the prevalence of upper limb (UL) weakness in acute stroke is approximately 70%, provided by data from the Copenhagen Stroke Study (n = 421) collected in 1991/92.^1^ Other studies from this era have provided even higher prevalence of UL weakness that ranged from 73-77%.^2-4^ It is possible that the overall prevalence and profile of UL weakness has changed since the above data were collected given advancements in the medical management of acute stroke. For instance, one large unselected study of first-ever stroke (n = 642) collected in 2009/10 in Sweden estimated only 48% of their sample had UL motor impairment.^5^ Screening data collected in 2017/2018 from an ongoing longitudinal study in Switzerland reported only 57% of consecutive patients with ischaemic stroke (n = 845) experienced UL weakness.^6^ It is interesting to ponder whether these more recent studies reflect a true change in UL weakness/motor impairment prevalence, differences in sample and/or study methods and/or the increasing use of reperfusion therapies. Capturing a true prevalence value is challenging. Selected arm weakness/impairment measure, threshold scores adopted, time of weakness assessment and number of sites data are collected from have the potential to make a large impact on the accuracy of prevalence estimation aiming to capture the true effect of stroke on the UL. Adding to the complexity is that rehabilitation researchers hoping to use prevalence values to inform clinical trial planning may be interested in prevalence studies that align with their population of interest, planned time post-stroke at recruitment and type of institution where their research will take place. The purpose of this study is to explore potential factors contributing to the accuracy of prevalence values of UL weakness using a case study from a single regional centre. Specifically, we used screening data for a prospective longitudinal cohort study to estimate UL weakness prevalence values using two different weakness measures at three different time points post-stroke to highlight the challenges of obtaining true prevalence values and/or values that may inform clinical trial planning.

## Methods

This case study used prospective screening data collected from a consecutive sample longitudinal cohort study examining UL recovery post-stroke.

### Population

All individuals with a suspected diagnosis of stroke admitted to the stroke unit of a regional hospital (Vancouver General Hospital) between February 28, 2016 and August 31, 2017 were screened for study inclusion. This regional hospital has a comprehensive tertiary stroke centre, serving the Vancouver city population of approximately 675 000. It is one of only three comprehensive centres in the province (population 4.6 million).^7^ While the vast majority of stroke patients are treated locally, approximately 14% of patients^8^ are transferred from other sites in Metro Vancouver which has a population of approximately 2.8 million.^7^ Another 10% of patients are transferred from health authorities outside of Metro Vancouver and would have required air ambulance. Data were collected from only those individuals with a diagnosis of ischaemic stroke or primary intracerebral haemorrhage confirmed by CT or MRI. Individuals with subarachnoid haemorrhage, cerebral venous thrombosis and those assessed greater than 7 days after their stroke (N = 35) or receiving palliative care (N = 11) were excluded. First-ever stroke admission was the primary unit of analysis in this study. Prior stroke was determined through physician history notes. Individuals whose chart was inaccessible were excluded as information regarding recurrent stroke was missing (N = 24).

### Procedures

Upper limb weakness was captured using the Shoulder Abduction and Finger Extension (SAFE) score. The SAFE score is the sum of the Medical Research Council (MRC) strength gradings (0-5) for shoulder abduction and finger extension (total range 0-10) and it is shown to be highly predictive of UL functional recovery after stroke.^9^ Physical therapists completed SAFE scoring unless circumstances prevented administration (ie. discharge and unwell). Upper limb weakness was defined as a SAFE score of 8 or less. This cut-off was selected based on the original PREP algorithm which states that a SAFE score of 8 or above is predictive of a full recovery, which is defined as the potential to return to normal or near-normal hand and arm function within 12 weeks.^10^ We chose a more conservative cut-off score of 8 for our longitudinal study because a SAFE score of 8 could represent the combination of normal strength (ie. 5/5) in one component and more significant weakness in the other component (ie. 3/5-antigravity movement without resistance). To reduce burden, therapists were allowed to indicate ‘no weakness’ on the SAFE forms if participants had a score greater than 8. Thus, SAFE scores of 9 or 10 were not always indicated on the form. Weakness categories were further defined as severe (0-4) and mild/moderate (5-8) and little to none (>8). In general, severe UL weakness refers to no active movement through to movement when gravity is eliminated, and mild/moderate weakness refers to movement against gravity with strength reduced.

Upper limb weakness was also captured retrospectively via chart review using the National Institutes of Health Stroke Scale (NIHSS) arm score indexed at admission and 24 hours post-admission. The NIHSS arm score captures weakness on a 5-point scale after asking patients to keep their arm raised against gravity for 10 seconds (90° in seated position and 45° in supine position). Scores range from 0 (no drift) to 4 (no movement). Weakness categories were further defined using the NIHSS arm score as severe (3-4) (no movement or effort against gravity); mild/moderate (1-2) (weak antigravity movement) and little to none (0). Demographic and clinical information (including length of stay and discharge disposition) were collected from patient medical records. Ethics approval was obtained from the local university and hospital review boards. Informed consent was not required as data were collected in the process of screening for a consecutive sample observation study.

### Statistical Analysis

Descriptive statistics were used to characterize the sample. All admission SAFE forms which indicated ‘no arm weakness’ were given a numerical score of 9. Half-ratings (ie. ‘+’or ‘−’) were ignored unless total numerical score was 8. One or more ‘+’half-rating with a score of 8 was considered ‘no arm weakness’. Distribution of weakness scores for the initial NIHSS arm scale was determined for individuals with SAFE and initial NIHSS arm scores. Distribution of weakness scores for the 24-hour post-admission NIHSS arm score was determined for individuals with completed SAFE, initial NIHSS and 24-hour post-admission arm scores. This was done to compare weakness category proportions in a consistent sample. All available baseline and 24-hour NIHSS arm scores were also analysed to determine the proportion of those with UL weakness using the full NIHSS arm score datasets.

## Results

**Figure 1.**
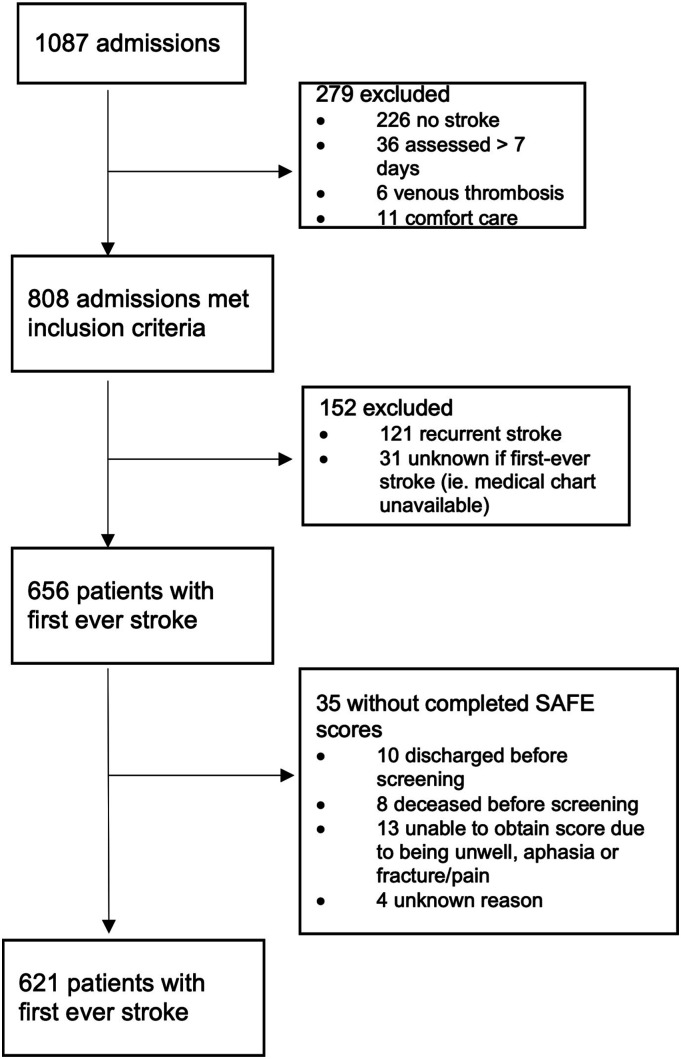
Summary of included admissions with Shoulder Abduction and Finger Extension Scale scores.

**Figure 2.**
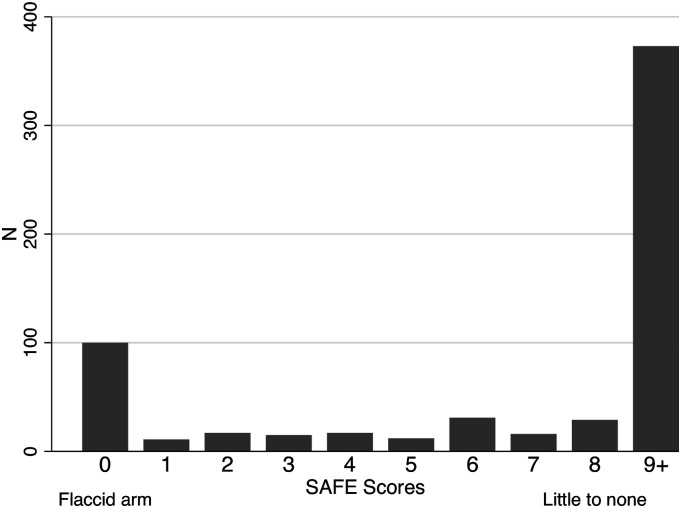
Distribution of Shoulder Abduction and Finger Extension Scale scores. Note: Severe 0-4; mild/moderate 5-8; little to none (>8).

A total of 1087 admissions were screened, and 808 admissions met the inclusion criteria (Figure 1). A total of 656 patients (575 ischaemic and 81 intracerebral haemorrhage) experienced first-ever stroke of which 621 (94.7%) were administered the SAFE at a mean of 1.9 days post-stroke (SD 1.5; range 0-7 days). Of those with SAFE scores, a NIHSS score of 1 or greater on the ‘arm weakness’ component of the scale was demonstrated by 56.6% at baseline and 48.9% at 24 hours post-admission. These numbers increased to 58.6% and 50.9%, respectively, when including all individuals with initial (N = 597) and 24-hour post-admission (N = 501) NIHSS arm scores. At the time of SAFE evaluation, 39.9% of patients with first-ever stroke had any UL weakness (SAFE ≤8) and 64.5% of those with UL weakness had severe weakness (Table 1). Severity of arm weakness was bimodal, with the majority having either little to no arm weakness (60.1%) or a flaccid arm (16.1%) (ie. SAFE score=0) (Figure 2). When recurrent stroke admissions were added to first-ever stroke admissions, data were very similar: 40.7% of 734 patients demonstrating UL weakness according to the SAFE, 56.4% of 648 patients according to the initial NIHSS arm score and 50.1% of 555 patients according to the 24-hour post-admission NIHSS arm score.

**Table 1.** Upper Limb Weakness Categories for Individuals with First-Ever Stroke, N (% of Total Patients; % of Patients With Weakness).

Weakness category	Initial NIHSS arm score (N = 546)^a^	24-hour NIHSS arm score (N = 454)^a^	SAFE score (mean 1.9 days post-stroke) (N = 621)^b^
Any weakness	309 (56.6%)	222 (48.9%)	248 (39.9%)
Severe (% total N; % with weakness)	148 (27.1%; 47.9%)	85 (18.7%; 38.3%)	160 (25.8%; 64.5%)
Mild/moderate (% total N; % with weakness)	161 (29.5%; 52.1%)	137 (30.2%; 61.7%)	88 (14.2%; 35.5%)
Little to none	227 (41.6%)	232 (51.1%)	373 (60.1%)

Abbreviations: NIHSS: National Institute of Health Stroke Scale; SAFE: Shoulder Abduction and Finger Extension Scale.

^a^Weakness categories according to the NIHSS arm score were defined as follows: Severe (3-4) (no movement or effort against gravity); mild/moderate (1-2) (weak antigravity movement) and little to none (0). Reported initial NIHSS arm scores are for patients with SAFE scores and reported 24-hour NIHSS arm scores are for patients with initial NIHSS arm scores and SAFE scores.

^b^Upper limb weakness according to the SAFE was defined as a score of 8 or less. Weakness categories according to SAFE were defined as follows: Severe (0-4) (no active movement to gravity-assisted movement); mild/moderate (5-8) (movement against gravity with strength reduced) and little to none (>8).^5^ N = 16 with missing SAFE form date of administration.

Importantly, 22.3% (N = 122) of individuals with initial NIHSS arm and SAFE scores had weakness according to the initial NIHSS arm score (NIHSS arm score >1) but were classified as having little to no weakness according to the SAFE (SAFE score >8). In addition, 5.7% (N = 31) of individuals with initial NIHSS arm and SAFE scores had no weakness according to the initial NIHSS arm score (NIHSS arm score = 0) but were classified as having weakness according to the SAFE (SAFE≤8).

Finally, Table 2 reports demographic, clinical and discharge destinations for the sample of individuals with SAFE scores by SAFE weakness category. Only 8% of individuals with severe weakness were discharged directly home in contrast to the majority of individuals with little to no weakness (73%). In addition, those with severe weakness made up 45% of the individuals transferred to another hospital and 12.5% of those with severe weakness died during their hospital stay. Over a third of the total sample with ischaemic stroke received thrombolysis and/or thrombectomy (37%). Interestingly, over half of individuals with severe UL weakness and ischaemic stroke received thrombolysis and/or thrombectomy (55%).

**Table 2.** Sample Demographic and Clinical Characteristics by SAFE Weakness Category.

Characteristic	Total N = 621^a^	Mild/moderate N = 88	Severe N = 160	Little or no weakness N = 373
Age mean (SD)	69.0 (14.6)	74.2 (14.1)	68.6 (15.6)	68.0 (14.1)
Female N (%)	262 (42.2%)	48 (54.5%)	65 (40.6%)	149 (39.9%)
Hemisphere affected N (%)				
Right	250 (40.2%)	44 (50.0%)	66 (41.2%)	143 (37.5%)
Left	307 (49.4%)	37 (42.0%)	84 (52.5%)	190 (49.9%)
Both	64 (10.3%)	7 (7.9%)	10 (6.2%)	48 (12.6%)
Type of stroke, N (%)				
Ischaemic	543 (87.4%)	80 (90.9%)	130 (81.2%)	333 (89.3%)
Haemorrhage	78 (12.6%)	8 (9.1%)	30 (18.7)	40 (10.7%)
Received thrombolysis and/or thrombectomy (% total, % ischaemic stroke)	199 (32.0%, 36.6%)	33 (37.5%, 41.2%)	71 (44.4%, 54.6%)	95 (25.5%, 28.5%)
Length of stay, mean (SD)	13.8 (15.4)	15.5 (15.1)	26.5 (23.9)	9.1 (10.5)
Discharge destination, N (%)				
Home	319 (51.4%)	35 (39.8%)	13 (8.1%)	271 (72.6%)
Rehab	131 (21.1%)	23 (26.1%)	60 (37.5%)	48 (12.9%)
Long-term care	37 (5.9%)	8 (9.1%)	17 (10.6%)	12 (3.2%)
Transferred to another hospital	110 (17.7%)	19 (21.6%)	50 (31.2%)	41 (11.0%)
Deceased	24 (3.9%)	3 (3.4%)	20 (12.5%)	1 (.3%)
Initial NIHSS,^b^ mean (SD)	7.8 (7.1)	8.6 (6.3)	14.4 (7.5)	5.3 (5.4)
24 hour NIHSS,^c^ mean (SD)	6.4 (6.9)	6.9 (4.4)	15.0 (6.9)	2.9 (3.6)

Abbreviations: NIHSS: National Institute of Health Stroke Scale; SAFE: Shoulder Abduction and Finger Extension Scale.

^a^N = 24 patients with inaccessible chart (ie. missing information about recurrent stroke).

^b^Initial NIHSS was the NIHSS performed in the ER by the stroke neurologist or the first NIHSS completed by the nurse in the stroke unit if the former was not completed; N = 482 patient charts accessed with all NIHSS initial items completed.

^c^24-hour NIHSS score which was the NIHSS completed by the nurse the next morning following admission; N = 432 patient charts accessed with all NIHSS 24-hour items completed.

## Discussion

This case study, which used screening data for a prospective longitudinal cohort study examining UL recovery, found that 40% of the 621 individuals administered the SAFE at a mean of 2 days post-stroke experienced UL weakness as defined by a SAFE score ≤8. Importantly, weakness data collected retrospectively using the admission NIHSS arm scores in our cohort resulted in a prevalence value almost 20% higher than that captured by the SAFE approximately 2 days later. Despite the differences in these values, both these proportions are less than the 69-77% from earlier studies^1-4^ except for the one 2009/10 published statistic of 48%.^5^ The 2017/2018 statistic of 57%^6^ which used the NIHSS arm score was very similar to our prevalence value using the full dataset of admission NIHSS arm scores (59%). These data may suggest that fewer people may experience UL weakness early after stroke compared to the 1990s. However, one should be aware of the limitations of these metrics. Our data show the potential effects that metrics and score cut-offs for characterizing weakness, time of weakness measurement and differences in sample characteristics can have on the accuracy of prevalence values. Indeed, variation in these above factors could potentially explain observed disparities in weakness prevalence across studies. Table 3 compares study methods and sample characteristics from seven studies that provide UL weakness/motor impairment prevalence values from unselected cohorts. The potential implications of the varied characteristics are discussed below.

**Table 3.** Comparison of Upper Limb Weakness/Motor Impairment Prevalence Articles.

	Kotila et al, 1984^a^	Nakayama et al, 1994	Lawrence et al, 2001	Rathore et al, 2002	Perssons et al, 2015	Held et al, 2019	Simpson et al (current dataset)
N	154	421	1259	474	642	845	621
Country	Finland	Denmark	UK	US	Sweden	Switzerland	Canada
Years data collected	1978-1980	1992-1993	1995-1998	1987-1997	2009-2010	2017-2018	2016-2017
% with impairment	73%	69%	77%	75.5%	48%	57%/46%^b^	40%
Time of UL assessment	Maximum impairment (≤24 hours)	≤7 days (median 13 hours)	Maximum impairment	Time of incident exam	<72 hours	Admission/screening^b^	≤7 days (mean 1.9 days)
Severity profile	NP	53% severe 47% mild/moderate	NP	NP	NP	NP	64% severe 36% mild/moderate
Measure of UL motor impairment	Weakness^c^	SSS arm and hand scores	Weakness^c^	Weakness^c^	MAS arm function, grip and pinch scores^d^	NIHSS arm score	SAFE score
Definition of weakness/impairment	NP	SSS arm and hand less than maximum	NP	NP	MAS (arm, grip or pinch scores) less than maximum	NIHSS arm score less than maximum	SAFE ≤8
Ischaemic/haemorrhagic^e^	NP	74.6%/4%	69%/19.7%	84.8%/15.2%	89.6%/10.4%	100%	87.4%/12.6%
Recurrent stroke (Y/N)	Y	Y	N	N	N	Y	N
Age (mean)	61 (17-90)	74.8 (11.2)	71.7 (14.2)	62.5 (6.1)	72.7 (14.2)	NP	69.0 (14.6)
Overall							
Stroke severity, mean (SD); median	NP	SSS^f^: 35.9 (17.4); NP	NP	NP	NIHSS^g^: 6.0 (6.8); 3	NIHSS^h^: NP; 5	NIHSS: 7.8 (7.1); 5
Reperfusion therapies: thrombolysis; thrombectomy^i^	NP	NP	NP	NP	9.9%; 3.8%	26.4%; 25.4%	30%; 16.6%
Multi-site^j^ (Y/N)	Y	N	Y	Y	Y	N	N

Abbreviations: NP: not provided; SSS: Scandinavian Stroke Scale; MAS: Motor Assessment Scale; NIHSS: National Institute of Health Stroke Scale; SAFE: Shoulder Abduction and Finger Extension Scale; NA: not applicable as before 1995; UL: upper limb.

^a^Excluded 101 individuals who died within 1 year of stroke.

^b^% with impairment are provided for admission and screening time points, respectively. 70% of screening assessments were within 48 hours. Mean or median time post-stroke at screening unknown.

^c^Specific weakness measure not reported.

^d^MAS arm function, grip and pinch scores were used to capture UL motor impairment in 80% of the sample; 20% of the sample used other standardized test of UL function in chart.

^e^The remaining percentage for ratios that do not add up to 100% make up the unknown classification.

^f^Values reported are for the full Copenhagen Stroke Study sample (N = 1197).^26^

^g^46% of the sample had missing total NIHSS scores.

^h^22% of the sample had missing total NIHSS scores.

^i^% who received thrombolysis and thrombectomy expressed as % of ischaemic strokes.

^j^Multi-site refers to studies that used data from more than one hospital.

### Weakness/Motor Impairment Measurement and Definition

Selection of UL weakness measure is an important decision in the design of a UL weakness prevalence study. Feasibility of measurement has to be weighed against the potential effect on sensitivity. The SAFE score was selected as the UL weakness screening measure for our longitudinal study due to its quick administration with our large cohort and its ability to predict functional recovery at various time points.^9-11^ The SAFE score used in our case study and the arm and hand component of the Scandinavian Stroke Scale (SSS) used in the Copenhagen Stroke Study only capture weakness in two components (ie. shoulder and hand). It is possible that critical motor impairment could exist in other movements captured by a multicomponent motor impairment measure such as the Motor Assessment Scale (MAS) or Fugl-Meyer. Interestingly, the SAFE, MAS and SSS arm and hand components are all strongly correlated to the Fugl-Meyer however and thus are likely picking up a similar construct of motor impairment.^9,10,12^ In addition, the authors of the PREP2 algorithm found that the SAFE and Fugl-Meyer had the same accuracy for predicting functional recovery at 3 months.^9^

The NIHSS arm score is a potentially convenient measure of UL weakness to capture weakness prevalence due to its widespread use. However, the NIHSS only measures weakness in one joint and only captures antigravity movement without resistance (MRC strength score 3). Prevalence as captured by the NIHSS arm scale used in our study (59%) and the Swiss study^6^ (57%) could therefore be underestimating the true prevalence of UL weakness at the time of hospital admission. Future studies comparing the correlation and predictive ability of the NIHSS arm scale and other multicomponent measures of weakness are thus warranted. Interestingly, three of the four early studies, apart from the Copenhagen Stroke Study, did not specify the weakness measure used. They were all accessed via the medical chart or through a medical exam.^2-4^ It is thus unknown whether they captured weakness at a single or multiple joints and whether they captured strength against resistance. Despite this lack of information concerning the precise UL measure used in the majority of the earlier studies, UL weakness prevalence from all four earlier studies fell within a range of 8% (ie. 69-77%).

The selection in weakness/motor impairment score cut-off is another important factor when considering the sensitivity of UL weakness prevalence values for capturing the full spectrum of weakness. All studies that specified the UL measure used, apart from our study, defined weakness/motor impairment as 1 point less than the measure’s maximum score. The low UL weakness prevalence found in our study could have been influenced by our weakness definition (ie. SAFE score <9 vs SAFE score <10). This selected cut-off was based on the PREP algorithm, which predicts that individuals with a SAFE score of 8 or more at 3 days post-stroke will achieve a full recovery at 3 months post-stroke. However, exclusion of this group of people has implications on the sensitivity of our prevalence value obtained. While individuals with a SAFE score of 9 may be able to produce mild resistance against gravity in either finger extension or shoulder abduction, this group still experiences weakness that may benefit from existing rehabilitation interventions. Importantly, our prevalence value would likely be substantively higher than 40% if our threshold of weakness included individuals with SAFE scores of 9. Unfortunately, we were not able to determine the number of individuals who had SAFE scores of 9 vs 10 because we allowed therapists to indicate ‘no weakness’ on the score forms if patients had scores above 8. Furthermore, it should be noted that a maximum score on the NIHSS arm score does also not necessarily equate to full strength as it does not measure strength against resistance. Thus, prevalence values based on a weakness definition of NIHSS arm score> 0 may not be capturing the full spectrum of weakness. Nonetheless, researchers planning future rehabilitation clinical trials that follow the recommendations by the international Stroke Recovery and Rehabilitation Roundtable to target individuals with more moderate to severe UL impairment^13^ may be interested in prevalence values that more closely reflect their population of interest.

Understanding the severity profile of UL weakness is useful for researchers planning future clinical trials that target specific severity groups. Our single-site study found that approximately one-third of people with UL weakness experienced mild/moderate weakness compared to approximately 50% in the Copenhagen Stroke Study. The SAFE score cut-off selected could have impacted the apparent increase in proportion of severe weakness observed in our study compared to the Copenhagen Stroke Study. The addition of the individuals with SAFE scores of 9 would increase the tally of individuals with mild weakness and may even up the proportion of those with severe/mild-moderate weakness. In addition, the proportion of individuals with severe stroke appeared to greatly increase when weakness was captured by the SAFE score compared to when captured by the 24-hour post-admission NIHSS arm scores. This could reflect any of the following: worsening of UL weakness, biased make-up of missing data in the 24-hour post-admission NIHSS arm scores (27% missing) or differences in how weakness is captured and categorized across the SAFE and NIHSS arm scales. This further highlights the complexity of comparing prevalence rates across different time points using different weakness measures. Our finding that a greater proportion of those with weakness experience severe weakness (as captured by the SAFE score) is consistent with a 2019 opinion article written by Hawe et al.^14^ This study compiled data from six studies^15-20^ (N = 373) exploring prediction of motor recovery captured by the Fugl-Meyer. The authors highlighted that the majority of initial Fugl-Meyer scores (captured <2 weeks post-stroke) in their compiled dataset was less than 12 points out of 66, which coincides with severe UL impairment.^21^ Furthermore, in our sample, we found that 62% of people within the severe category had a SAFE score of 0. This is concerning given the dearth of evidence-based rehabilitation treatments for people with severe UL impairment and our finding that these individuals are least likely to go home.^22^ Importantly, interventions are needed for this population to help move them towards a more moderate level of impairment so that they too can participate in and benefit from intensive therapy programmes that involve high doses of active movement repetitions. Furthermore, future studies may determine whether the distribution of UL weakness (in our case, bimodal) is related to factors such as corticospinal tract damage or psychometrics of the measurement scale (eg. floor/ceiling effects).

Finally, discharge data from our single centre may highlight potential challenges for researchers conducting UL rehabilitation trials in other comprehensive stroke centres trying to recruit individuals with severe weakness. Approximately 44% of individuals with severe weakness in our case study were either transferred to another hospital or died before discharge making recruitment of this population into rehabilitation trials very challenging. Multiple sites may be necessary to complete acute rehabilitation clinical trials for individuals with severe impairment in a timely manner.

### Time of Weakness/Motor Impairment Measurement

There was variation across studies in the time weakness/motor impairment was assessed. Two of the earlier studies captured weakness at the time of maximum deficit and therefore would likely obtain higher prevalence values in their sample than if they captured weakness at a fixed time point.^2,4^ Nakayama et al,^1^ Rathore et al^4^ and Held et al^6^ captured weakness at admission to the hospital, and thus, the majority of cases are presumed to have been assessed within 24 hours. Importantly, later time points in which weakness/motor impairment was assessed in more recent studies were likely a significant factor for observed differences in weakness prevalence values between earlier and more recent studies. Persson et al^5^ and our study captured weakness/motor impairment at a slightly later time point of 1.5 days post-admission and 1.9 days post-stroke, respectively, which reflects weakness after acute reperfusion therapies have been administered. The potential time difference in weakness assessment of a day or two is not a trivial one. For instance, the prevalence value of 57% found in the Held et al^6^ and captured at admission to the hospital decreased to 46% captured at a screening evaluation where 70% of evaluations took place within 48 hours of stroke. In addition, we found a prevalence rate of 57% using the initial NIHSS arm score, but this value decreased to 40% using the SAFE when captured at a mean time of 1.9 days post-stroke in the same sample. Moreover, the 153 individuals classified differently according to the initial NIHSS arm task vs SAFE could represent true recovery or deterioration across the time in between these 2 measures’ administration or could represent differences in how weakness is measured and defined. This point highlights the importance of including a time qualifier when citing UL prevalence values. Indeed, the inclusion of a time qualifier allows consumers of prevalence data to gauge which information is most helpful for them. For instance, researchers hoping to conduct clinical trials in the acute phase may want to know rates of weakness closer to the time they are trying to recruit participants. Furthermore, future studies that use the same weakness measure in a non-selected sample and capture weakness at very fine intervals within the first week post-stroke are needed to examine the time course of UL weakness prevalence early after stroke.

### Sample Characteristics

Differences in sample characteristics could also contribute to the accuracy of arm weakness/motor impairment prevalence and differences in observed statistics across studies. Exclusion of individuals based on the inability to access them (ie. due to early discharge) or difficulty assessing (ie. due to severe aphasia or other medical issues) can impact the sensitivity of prevalence estimates. Indeed, our case study excluded individuals who were assessed using the SAFE greater than 7 days post-stroke (N = 36) and those whose SAFE score could not be obtained due to early death, being unwell or having severe aphasia (N = 18). It is possible that these individuals had severe UL stroke which could result in the underestimation of the true prevalence of UL weakness as captured by the SAFE assessment in our study. Importantly, this appears to be a challenge not unique to our case study. For instance, the Copenhagen Stroke Study reported N = 44 individuals unable to assess at admission^1^; Lawrence et al^3^ reported N = 39 individuals unable to assess at the time of maximum impairment and Kotila et al^2^ reported excluding 101 individuals who died within the study period. This issue further highlights the difficultly of including people with severe stroke in research studies.

Upon visual inspection, the variation in the mean age does not appear to form a pattern in those studies finding high or lower prevalence values. The inclusion or exclusion of individuals with haemorrhagic stroke could potentially explain some of the differences in prevalence across studies. Six of the seven reported statistics included individuals with haemorrhagic stroke whereas the 2017/2018 Swiss data^6^ only included ischaemic stroke. One might expect weakness prevalence in studies that excluded haemorrhagic stroke to be lower than studies that included them.

The distribution of overall stroke severity could also impact observed UL weakness prevalence. The level of stroke severity in our study, as captured by the NIHSS, was similar to that of Held et al.^6^ Both of these studies found similar UL weakness prevalence captured by the NIHSS arm scale at admission to their respective hospitals (ie. 59% and 57%). The median stroke severity in the study of Persson et al^5^ was lower. However, their reported value should be treated with caution due to the large percentage of missing total NIHSS scores (46%). It is possible that greater public awareness of stroke symptoms and the improved recognition of minor stroke/TIA as a neurological emergency has led to greater numbers of people with mild stroke being admitted to the hospital in recent years.^23^ Interestingly, we did not find a large difference in proportion of mild strokes between our study and the Copenhagen Stroke Study, which is the only earlier study to report overall stroke severity of the sample. In the latter, 40% had a mild stroke as defined by the SSS, while we found 35% of our cohort had a mild stroke after converting NIHSS initial scores to SSS scores (N = 482).^24^ While the expert care of the comprehensive stroke centre from our study might potentially contribute to lower prevalence of UL weakness, the nature of comprehensive stroke centres with 24/7 access to stroke neurologist specialists often results in a more complex stroke patient population. Moreover, as difficult cases are transferred from surrounding areas, the additional transfer time would contribute to worse outcomes.

The rapid administration of reperfusion therapies for individuals with ischaemic stroke has greatly advanced stroke care. It is difficult to determine the effect of reperfusion therapies on UL weakness in the absence of randomized control trials that include specific UL weakness measures. The apparent decrease in UL weakness found in more recent studies could be largely due to the later time point at which weakness was captured or could be due to other differences in study and sample characteristics. Nonetheless, it is reasonable to expect that rates of weakness/motor impairment might be lower after receiving these therapies compared to rates at admission due to the fact that reperfusion therapies have become standard of care due to their reported effects on disability. The prevalence of UL weakness/motor impairment after any reperfusion therapies have been administered is arguably the most relevant to researchers and healthcare administrators as rehabilitation services and clinical trials conducted in the acute phase will most certainly be starting after reperfusion therapies have been administered. Nonetheless, more studies are needed to capture prevalence both at admission and within a few days post-stroke to determine how or if UL weakness prevalence is changing. In addition, thrombolysis rates in comprehensive stroke centres have previously been shown to be much higher than those observed in primary stroke centres.^25^ Thus, any potential effect of reperfusion rates on our observed UL weakness prevalence may not generalize to non-comprehensive stroke centres.

Finally, more accurate estimations of UL weakness prevalence on the population level are obtained from multi-site data. Estimates obtained from single sites such as those from our study, the Copenhagen Stroke Study^1^ and the Held et al^6^ study may not generalize to the greater population of individuals with stroke. The increasing collection of multi-centred stroke databases (eg Greater Cincinnati/Northern Kentucky Stroke Study) may provide more accurate estimates in the future.

## Conclusion

This case study observed a lower rate of UL weakness (40% and 59%) than expected. A variety of factors may have contributed to our lower values such as exclusion of individuals with mild weakness, exclusion of some individuals with severe stroke, weakness captured at a later time post-stroke and high reperfusion rates and highlights the challenges of obtaining accurate population-level estimates of UL weakness prevalence early after stroke. Researchers using UL prevalence data to inform clinical trial planning should consider differences between their planned time of recruitment and UL impairment inclusion criteria to those used in prevalence studies. They should also consider the added challenges of recruiting individuals with severe stroke and consider multiple sites to support the important investigation of new interventions aimed at individuals with severe UL weakness.
